# Automated deep-learning quantification of nine patellofemoral instability parameters on multislice CT images: development and validation of the GU2Net model

**DOI:** 10.1302/2633-1462.73.BJO-2025-0326.R1

**Published:** 2026-03-12

**Authors:** Qin Ye, Yingying Ying, Jiake Hua, Junfen Ye, Chengxin Zhu, Bowen Zheng

**Affiliations:** 1 Center for Rehabilitation Medicine, Department of Radiology, Zhejiang Provincial People’s Hospital, Affiliated People’s Hospital, Hangzhou Medical College, Hangzhou, Zhejiang, China; 2 Department of Radiology, The People’s Hospital of Pingyang, Wenzhou, China; 3 The Second School of Clinical Medicine, Hangzhou Normal University, Hangzhou, Zhejiang, China; 4 Taizhou Integrated Traditional Chinese and Western Medicine Hospital, Taizhou, Zhejiang, China; 5 Department of Radiology, Tongxiang First People's Hospital, Jiaxing, Zhejiang, China

**Keywords:** Deep learning, Patellar instability, CT, patellofemoral instability, trochlear groove, knees, tibial tubercle, intraclass correlation coefficient (ICC), sulcus angle (SA), lateral patellar tilt, transepicondylar axis, Pearson correlation coefficient

## Abstract

**Aims:**

Objective and precise measurement of patellar instability (PI) parameters on CT images is essential for accurate diagnosis and treatment planning. However, manual assessment is tedious, time-consuming, and prone to error. This study aimed to develop and validate a deep learning model that automatically quantifies PI parameters on axial knee CT images.

**Methods:**

CT scans of 1,125 knees were randomly divided into training, validation, internal test, and hold-out test sets. A deep learning-based model was trained to localize anatomical landmarks and calculate nine PI parameters: lateral patellar tilt (LPT), bisect offset ratio (BSO), congruence angle (CA), sulcus angle (SA), trochlear groove depth (TGD), lateral trochlear inclination (LTI), trochlear groove-transepicondylar axis (TG-TEA) distance, tibial tubercle-trochlear groove (TT-TG) distance, and tibial tubercle-Roman arch (TT-RA) distance. Model performance was compared with manual measurements using the successful detection rate, mean absolute error (MAE), intraclass correlation coefficient (ICC), and Pearson correlation coefficient.

**Results:**

The model accurately predicted landmark locations (MAE 0.84 to 2.72 mm) and PI parameters (ICC 0.826 to 0.997, *r* 0.705 to −0.994, p < 0.001) except for SA (ICC 0.701 to 0.862, *r* 0.542 to 0.744, p < 0.001). On the hold-out test set, the model outperformed inexperienced radiologists for LPT, CA, SA, LTI, and TGD (model: ICC 0.701 to 0.996, r 0.542 to 0.992, p < 0.001; radiologists: ICC 0.413 to 0.959, r 0.281 to 0.923, p* *< 0.05).

**Conclusion:**

The proposed deep learning model reliably automates PI measurement, reducing the time and variability associated with manual assessment and mitigating dependence on examiner experience.

Cite this article: *Bone Jt Open* 2026;7(3):357–365.

## Introduction

Patellar instability (PI) is a multifactorial condition that may lead to abnormal patellar tracking, functional limitations, and early osteoarthritis.^1,2^ More than 80% of patients with PI have at least one risk factor, including a lateralized tibial tubercle, trochlear dysplasia, patella alta, genu valgum, or rotational malalignment.^3^ Identifying these anatomical factors is critical for individualized management. Medial patellofemoral ligament reconstruction is recommended for recurrent instability without malalignment or trochlear dysplasia; tibial tubercle osteotomy is indicated for a lateralized tibial tubercle; and trochleoplasty is reserved for severe trochlear dysplasia.^1,4-6^

Reliable evaluation of PI depends on accurate measurement of PI parameters on CT images. These parameters include 1) lateral patellar tilt (LPT), bisect offset ratio (BSO), and congruence angle (CA) for patellar position;^7-10^ 2) sulcus angle (SA), trochlear groove depth (TGD), lateral trochlear inclination (LTI), and trochlear groove-transepicondylar axis (TG-TEA) distance for trochlear morphology;^11-14^ and 3) tibial tubercle-trochlear groove (TT-TG) distance and tibial tubercle-Roman arch (TT-RA) distance for a lateralized tibial tubercle.^15-17^ Recent guidelines clarify PI surgical thresholds (e.g. TT-TG > 20 mm), and mis-measurement can mislead surgeons into choosing inappropriate treatment plans.^1,4^

However, for radiologists and orthopaedic surgeons, obtaining these measurements is tedious, time-consuming, and prone to error because it requires identifying numerous subjective anatomical landmarks.^15,18-21^

Deep learning (DL) excels at detecting landmarks and objects in images, offering the potential to mitigate the limitations described above.^22-28^ Several DL-based methods for PI measurement have already been explored. Our group,^25^ along with Tuya et al^26^ and Barbosa et al,^27^ developed DL models that automatically quantify PI parameters by localizing landmarks on lateral radiographs, the Laurin view, and MRI, respectively. Although CT is the reference modality for PI assessment, existing DL models for CT images are limited to single-slice analyses and trochlear morphology parameters.^28^ Accordingly, this study aimed to develop and validate a DL model that computes a comprehensive set of PI parameters from multislice axial CT images.

## Methods

### Dataset collection

This retrospective study was conducted in accordance with the ethical guidelines of the Declaration of Helsinki^29^ and approved by the Ethics Committee of Zhejiang Provincial People’s Hospital (QT2025166). The requirement for informed consent was waived because only retrospective image data were used. Knee CT scans acquired between July 2021 and October 2023 were retrieved from the Picture Archiving and Communication System (Mindray, China). Images were obtained with a slice thickness of 1.0 mm to 5.0 mm, a 512 × 512 matrix, and 120 kVp on six CT scanners from three manufacturers (Siemens Healthcare, Germany; United Imaging Healthcare, China; and Philips Healthcare, USA). Exclusion criteria were prior surgery, fracture, malformation, poor image quality (knee flexion, insufficient image range, or blurred landmark), or knee overlap.

**Fig. 1:**
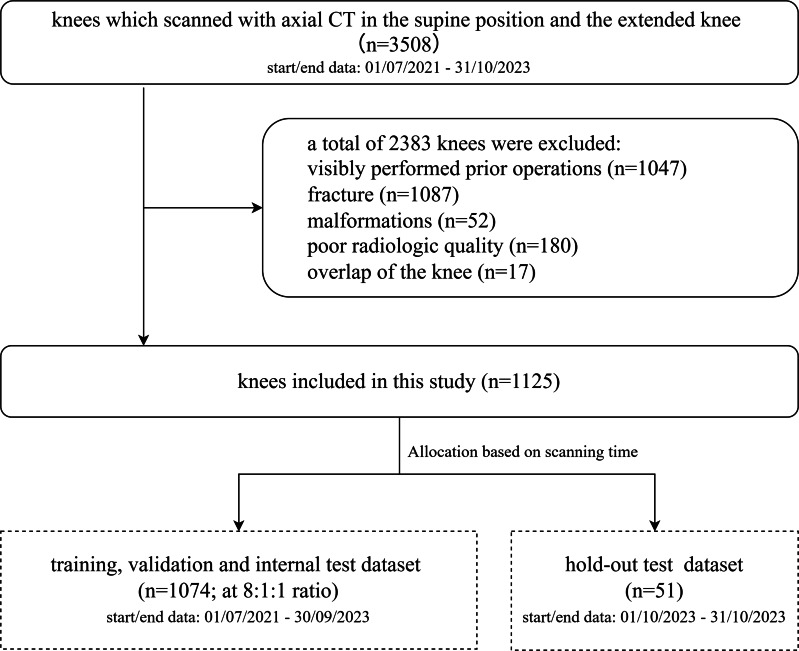
Flowchart of dataset selection.

A total of 2,383 knees were excluded from the study, while 1,125 eligible knees were included. Scans from the final month of the study period (n = 51) were reserved as the hold-out test set. The remaining 1,074 knees were randomly divided into training (n = 860), validation (n = 107), and internal (n = 107) test sets in an 8:1:1 ratio. Figure 1 shows the dataset selection flowchart.

The mean age was 44.25 years (10 to 96; SD 19.46) in the training/validation set, 42.63 years (11 to 87; 17.40) in the internal test set, and 41.31 years (13 to 76; 17.44) in the hold-out test set. All datasets had balanced laterality distributions (Table I).

**Table I.** Summary of dataset distribution.

Variable	Training and validation	Internal test	Hold-out test	p-value
No. of knees	967	107	51	
Mean age, yrs (SD)	44.25 (19.46)	42.63 (17.40)	41.31 (17.44)	0.426*
**Sex, n (%)**				0.058†
Male	440 (45.5)	39 (36.4)	17 (33.3)	
Female	527 (54.5)	68 (63.6)	34 (66.7)	
**Side, n (%)**				0.579†
Left	491 (50.8)	49 (45.8)	27 (53.8)	
Right	476 (49.2)	58 (54.2)	24 (46.2)	

* Analysis of variance.

† Chi-squared test.

### Image selection and annotations

Four radiologists with varying levels of experience participated in the study: two musculoskeletal experts (> 15 years each; E1, DH and E2, JFY), one first-year resident (R1, JKH), and one general radiologist (five years; R2, QY). All radiologists were blinded to clinical information and model prediction.

Before annotation, each radiologist received verbal and hands-on training with five knee CT scans not included in the study, using the LabelMe tool (version 5.0.0; GitHub, USA).

CT slices that showed the intact patella, the posterior condyles with the Roman arch, or the tibial tubercle at the first full insertion of the patellar tendon were selected and annotated by R1 and R2. All annotations were then reviewed and, when necessary, corrected by E2 to serve as the ground truth. Three landmarks were on the patella, eight on the femoral condyles, and one on the tibial tubercle.^30^ A detailed description of the landmarks appears in Table II.

**Table II.** Name and detailed description of landmarks.

Body	Name	Landmark description
Patella	A	Most medial part of the medial side of the patella
	B	Most posterior part of the articular surface of the posterior side of the patella
	C	Most lateral part of the medial side of the patella
Femur	D	Most anterior part of the lateral trochlear groove
	E	Lateral apex of the lateral femoral epicondylar prominence
	F	Most posterior part of the lateral femoral condyle
	G	Most posterior part of intercondylar femoral notch
	H	Most posterior part of the medial femoral condyle
	I	Medial apex of the medial femoral epicondylar prominence
	J	Most anterior part of the medial trochlear groove
	K	Deepest part of the trochlear groove
Tibia	T	Most anterior part of the tibial tubercle

**Fig. 2:**
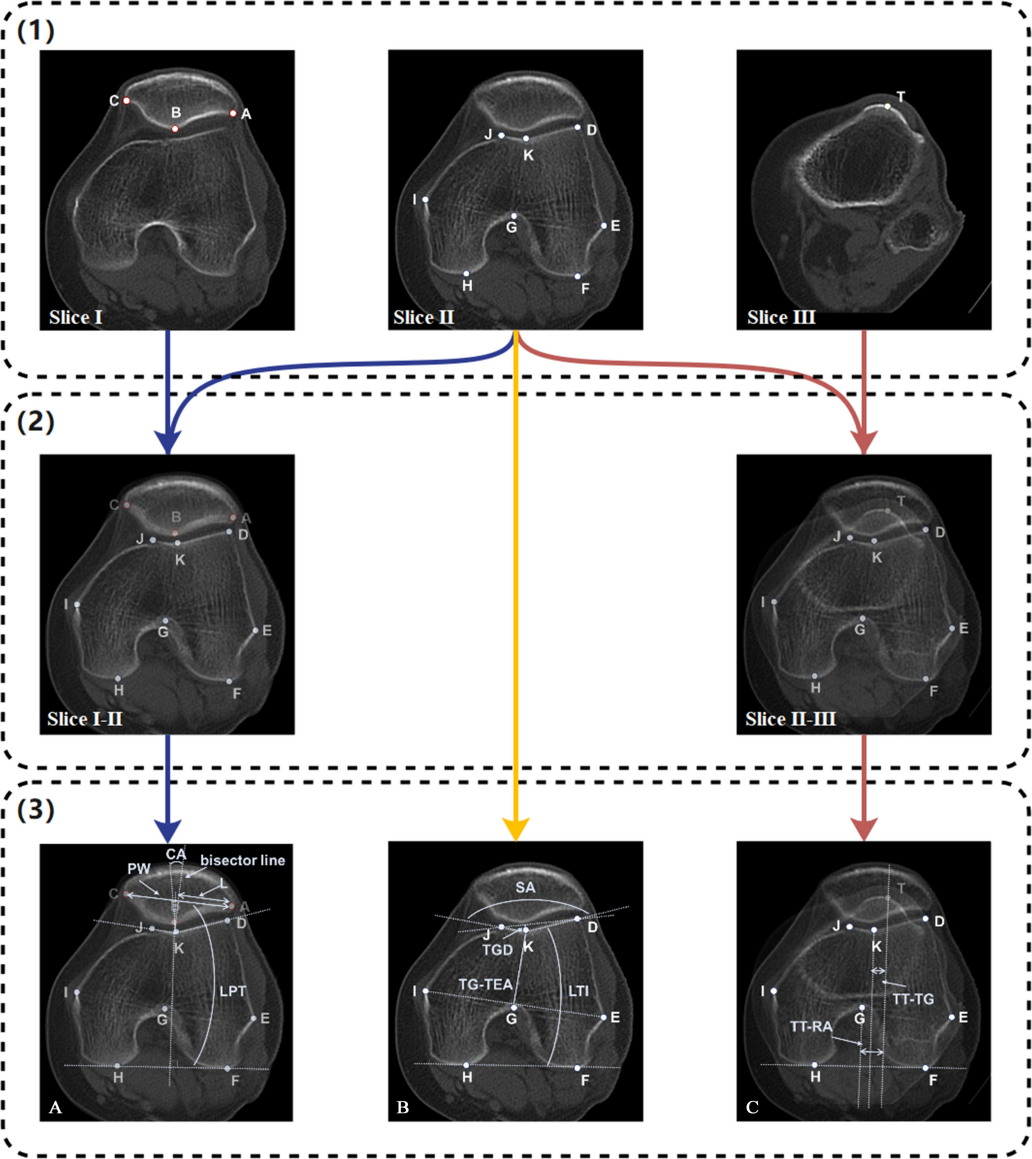
Patellar instability parameter measurement. (1) Landmarks A to C, landmarks D to K, and landmark T were located on the patella, the femoral condyle, and on the tibial tubercle, respectively. (2) Parameters related to patellar position, trochlear morphology, and tibial tubercle lateralization were determined on Slices I to II, Slice II, and Slices II to III, respectively; Slices I to II were obtained by superimposing Slices I and Slice II, and similarly, Slices II to III was obtained by superimposing Slices II and Slice III. (3 A) Lateral patellar tilt (LPT) is the angle of Line AC and Line FH. Patellar width (PW) is divided into two parts by a line perpendicular to Line FH through Point K, and the lateral part is named L; Bisect offset index (BSO) = L/PW × 100%. Congruence angle (CA) =$∠BKJ-\frac{1}{2}∠JKD$. (3B) sulcus angle (SA)=∠JKD. Lateral trochlear inclination (LTI) is the angle of Line KD and Line FH. Trochlear groove depth (TGD) is the perpendicular distance from Point K to Line FH. TG-TEA is the distance between Point K projected onto Line EI. (3 C) The tibial tubercle-to-trochlear groove distance (TT-TG) is the distance between Point K and Point T projected onto Line FH. The tibial tubercle-Roman arch distance (TT-RA) is the distance between Point G and Point T projected onto Line FH.

All four radiologists also manually measured the PI parameters in the hold-out test set, including LPT, BSO, CA, SA, TGD, LTI, TG-TEA, TT-TG, and TT-RA (Figure 2).

### Model implementation

The GU2Net (Global-Local U² Network) is a dual-path convolutional neural network designed to localize anatomical landmarks on knee CT images accurately.^31^ It integrates detailed local features with broader contextual information to improve landmark detection accuracy. The model consists of two parallel processing branches: a local branch and a global branch.

The local branch uses a U-Net–style encoder-decoder architecture to capture fine-grained spatial details. This design efficiently processes high-resolution input images. Through four down-sampling blocks, the encoder reduces image resolution while increasing feature complexity; the decoder then reconstructs the spatial resolution through up-sampling. The output is a set of heat maps indicating the probable locations of the 12 anatomical landmarks.

The global branch processes a lower-resolution version of the input image to incorporate wider contextual information. This helps the model better understand the overall anatomical structure, particularly in challenging cases or when landmarks are ambiguous. The output is then up-sampled to match the original image resolution.

**Fig. 3:**
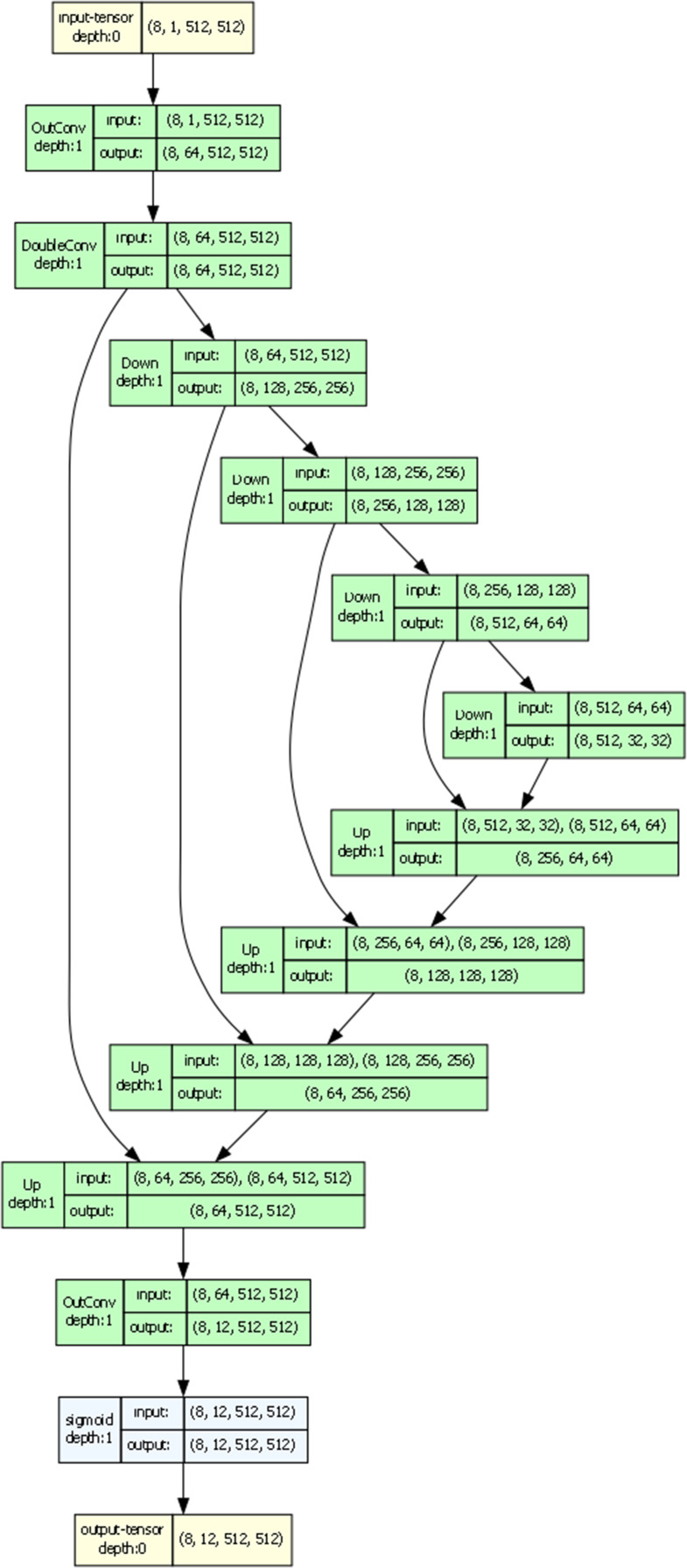
Global-Local U² network.

The model was trained for 100 epochs with a batch size of eight and a learning rate of 0.0001. The model was implemented using the PyTorch framework (version 3.11) and trained on a single NVIDIA GeForce RTX 4090 GPU (24 GB memory). Finally, the outputs from both branches are combined to produce the final landmark predictions (Figure 3). These predicted landmark coordinates were used with established mathematical formulae to automatically calculate the nine PI parameters on the most relevant CT slices, namely those showing the greatest patellar width or the greatest transepicondylar length.^32^

### Evaluation and statistical analysis

Statistical analyses were conducted in Microsoft Excel (version 16.61; Microsoft, USA) and SPSS Statistics (v. 26, IBM, USA). Normality was assessed for all variables, and p < 0.05 was considered statistically significant. Model performance was assessed on the internal and hold-out test sets. Landmark detection performance was quantified by the successful detection rate (SDR) and the mean absolute error (MAE (SD)) between model predictions and ground truth. The agreement between PI parameters generated by the model and those measured by radiologists of different experience levels was evaluated with the intraclass correlation coefficient (ICC) (2,1) and the Pearson correlation coefficient. An ICC ≥ 0.90 was deemed excellent, and an ICC ≥ 0.75 was considered good.^33^

## Results

### Performance of landmark detection

**Fig. 4:**
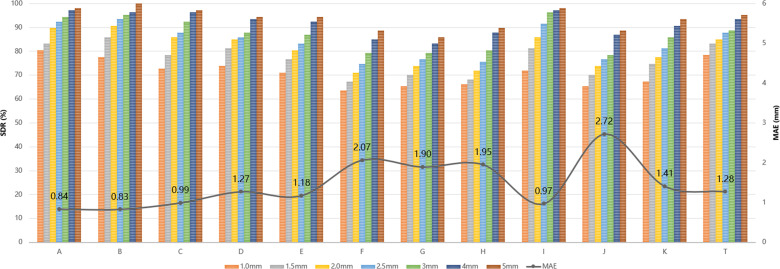
Graphic representation of the successful detection rate (SDR) and mean absolute error (MAE) of each landmark.

**Fig. 5:**
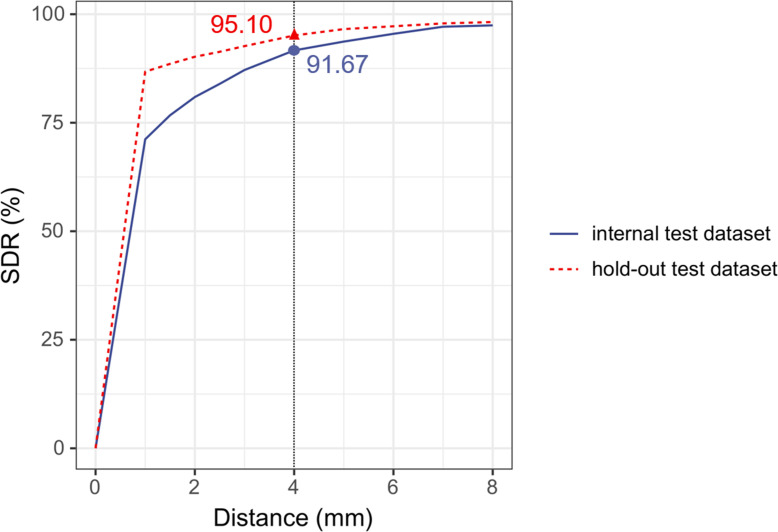
Overall performance of landmark detection. SDR, successful detection rate.

In the internal test set, the lowest MAE was recorded for Landmark A (0.84 mm (SD 1.07)) and the highest for Landmark J (2.72 mm (SD 8.81)) (Figure 4). The SDR within a 4 mm threshold was 91.67% in the internal test set and 95.10% in the hold-out test set (Figure 5).

### Performance of measurement prediction

Agreement between the two experts was excellent for all PI measurements (ICC > 0.90), and their values were used as the reference standard. The reliability of the model, R1 and R2, relative to this standard is summarized in Table III. The model achieves expert-level performance in measuring LPT, BSO, CA, LTI, TGD, TG-TEA, TT-TG, and TT-RA parameters, except for SA.

**Table III.** Ability of the model and each radiologist to measure patellar instability-related parameters.

Variable	Internal test dataset	Hold-out test dataset						
	Model vs groud truth	E1 vs E2	M vs E1	M vs E2	R1 vs E1	R1 vs E2	R2 vs E1	R2 vs E2
**LPT**								
ICC	0.992*	0.978*	0.973*	0.996*	0.870	0.870	0.959*	0.948*
r	0.983	0.956	0.947	0.992	0.798	0.795	0.923	0.903
p-value†	< 0.001	< 0.001	< 0.001	< 0.001	< 0.001	< 0.001	< 0.001	< 0.001
**BSO**								
ICC	0.992*	0.984*	0.980*	0.987*	0.949*	0.954*	0.979*	0.981*
r	0.984	0.970	0.962	0.975	0.906	0.917	0.959	0.964
p-value†	< 0.001	< 0.001	< 0.001	< 0.001	< 0.001	< 0.001	< 0.001	< 0.001
**CA**								
ICC	0.960*	0.910*	0.869	0.934*	0.629	0.622	0.826	0.736
r	0.924	0.835	0.770	0.877	0.463	0.454	0.707	0.583
p-value†	< 0.001	< 0.001	< 0.001	< 0.001	0.001	0.001	< 0.001	< 0.001
**SA**								
ICC	0.862	0.901*	0.701	0.796	0.439	0.413	0.749	0.736
r	0.774	0.822	0.542	0.669	0.296	0.281	0.600	0.587
p-value†	< 0.001	< 0.001	< 0.001	< 0.001	0.035	0.046	< 0.001	< 0.001
**TGD**								
ICC	0.877	0.914*	0.826	0.914*	0.668	0.641	0.825	0.802
r	0.792	0.844	0.705	0.847	0.517	0.495	0.710	0.685
p-value†	< 0.001	< 0.001	< 0.001	< 0.001	< 0.001	< 0.001	< 0.001	< 0.001
**LTI**								
ICC	0.947*	0.961*	0.900*	0.938*	0.745	0.774	0.871	0.889
r	0.907	0.925	0.823	0.886	0.627	0.677	0.773	0.805
p-value†	< 0.001	< 0.001	< 0.001	< 0.001	< 0.001	< 0.001	< 0.001	< 0.001
**TG-TEA**								
ICC	0.994*	0.993*	0.992*	0.997*	0.927*	0.947*	0.988*	0.988*
r	0.989	0.987	0.984	0.994	0.866	0.9	0.976	0.975
p-value†	< 0.001	< 0.001	< 0.001	< 0.001	< 0.001	< 0.001	< 0.001	< 0.001
**TT-TG**								
ICC	0.982*	0.989*	0.991*	0.991*	0.945*	0.931*	0.980*	0.988*
r	0.964	0.978	0.984	0.982	0.899	0.877	0.962	0.976
p-value†	< 0.001	< 0.001	< 0.001	< 0.001	< 0.001	< 0.001	< 0.001	< 0.001
**TT-RA**								
ICC	0.991*	0.992*	0.989*	0.994*	0.962*	0.961*	0.984*	0.978*
r	0.983	0.984	0.979	0.99	0.928	0.927	0.968	0.957
p-value†	< 0.001	< 0.001	< 0.001	< 0.001	< 0.001	< 0.001	< 0.001	< 0.001

* ICCs ≥ 0.9 indicate excellent reliability.

† Calculated using Pearson correlation coefficient.

BSO, bisect offset ratio; CA, congruence angle; E, expert; ICC, intraclass correlation coefficient; LPT, lateral patellar tilt; LTI, lateral trochlear inclination; R, radiologist; SA, sulcus angle; TGD, trochlear groove depth; TG-TEA, trochlear groove-transepicondylar axis; TT-RA, tibial tubercle-Roman arch distance; TT-TG, tibial tubercle-trochlear groove distance.

Except for SA, the model showed good to excellent agreement in both test sets. For BSO, TG-TEA, TT-TG, and TT-RA, agreement for the model, R1, and R2 was comparable. For LPT and SA, model performance matched that of R2, and surpassed that of R1. For CA, TGD, and LTI, the model achieved higher reliability than both R1 and R2.

## Discussion

In this study, we developed a deep-learning model to automatically detect PI-related landmarks and calculate the corresponding parameters on axial knee CT images. The model accurately localized the relevant landmarks; achieved performance comparable to musculoskeletal experts for LPT, BSO, CA, LTI, TGD, TG-TEA, TT-TG, and TT-RA; and outperformed less experienced radiologists for LPT, CA, SA, TGD, and LTI.

In our cohort, the largest MAE was 2.72 mm for Landmark J, and the overall SDR exceeded 90% at the 4 mm threshold, similar to or better than those reported in previous studies.^28,34^ The SDR at the same threshold exceeds 50% in the Aligner Model and 90% in the Patch Model.^28^ The strength of this study is the fusion of high-resolution detail from the local branch with context-aware features from the global branch.^31^ Accurate landmark detection provides a solid foundation for reliable PI measurement.

We also compared manual measurements obtained by radiologists with different levels of experience. The least experienced reader (R1) showed the lowest ICCs, whereas agreement between the two experts exceeded 0.90, indicating that accurate PI measurement requires substantial experience. Similar experience-related variability has been reported by Smith et al^20^ and Nicolaas et al.^21^ To mitigate this limitation, we developed a DL model that predicted LPT, BSO, CA, LTI, TGD, TG-TEA, TT-TG, and TT-RA with expert-level reliability. Moreover, the model surpassed inexperienced readers for LPT, CA, TGD, and LTI. Its reliability also exceeded the average values reported for manual measurements in the literature.^10-12,17,18^ These findings suggest that the proposed model can substantially assist radiologists – particularly those with limited experience – in obtaining precise PI measurements.

The model performed worst for SA, failing to match expert performance. Unlike our previous study,^25^ the current dataset included cases with osteoarthritis and was therefore more representative of the population. The reduced accuracy for SA may reflect osteophyte formation at landmark J – the anteromedial ridge of the trochlear groove – which complicates precise localization. Notably, the model’s ICC values for SA were comparable with those of R2 and higher than those of R1, indicating that, although the model did not reach expert level, it reduced the significant variability observed in less experienced radiologists.

Several groups have developed DL models for automated landmark localization and PI measurement.^25-28^ On plain radiographs, these methods automatically assessed patellar height, trochlear dysplasia, patellar tilt, and lateralization.^25,26^ Using a single axial or sagittal slice, other DL approaches evaluated trochlear dysplasia and patellar height.^27,28^ Compared with these state-of-the-art studies, our model provided a more comprehensive PI assessment and addresses the previously unmet need for automatic evaluation of tibial tubercle lateralization. Moreover, whereas prior models operated only on 2D images or a single slice from 3D data,^25-28^ our method realizes the measurement of patellar position and tibial tubercle lateralization across stacked axial CT slices.

This study has several limitations. First, although pathological CT images were included, the single-centre, relatively small sample size limited the model’s robustness. Incorporating larger, multicentre datasets during training is expected to improve performance. Second, this method addresses most of the alignment factors around the knee but remains incomplete without measuring patellar height, femoral anteversion, knee rotation, tibial torsion, and overall coronal or sagittal alignment. Third, most clinicians rely on MRI rather than CT to avoid irradiation in younger patients and to obtain more information on soft-tissues and articular cartilage. Finally, in this study, CT images containing structures relevant to PI parameters were selected manually, whereas clinical knee CT series are far more extensive. Future work should therefore include a classification model that automatically identifies PI-related images.

In conclusion, a deep-learning-based model for automatic PI measurement was developed. In its current form, the model predicts LPT, BSO, CA, TGD, LTI, TG-TEA, TT-TG, and TT-RA with expert-level accuracy, outperforming inexperienced radiologists for LPT, CA, SA, TGD, and LTI on axial CT images. Consequently, it substantially streamlines this labour-intensive task and reduces measurement variability attributable to operator experience.

**Take home message**

- Deep learning can be utilized for the automatic measurement of patellofemoral instability parameters on multislice CT images, thereby reducing the time and variability associated with manual assessment.

- The deep learning-based model achieves comparable performance to musculoskeletal experts in measuring lateral pateller tilt, bisect offset ratio, congruence angle, trochlear groove depth, lateral trochlear inclination, trochlear groove-transepicondylar axis, tibial tubercle-trochlear groove, and tibial tubercle-Roman arch.

## Data Availability

The data that support the findings for this study are available to other researchers from the corresponding author upon reasonable request.
